# Lectures on Physical Diagnosis

**Published:** 1845-05

**Authors:** J. R. Buck

**Affiliations:** Lecturer on the Theory and Practice of Medicine in the Louisville Summer School of Medicine


### WESTERN JOURNAL

OF

### MEDICINE AND SURGERY.

MAY, 1845.

Art. I.—*Lectures on Physical Diagnosis.* By J. R. Buck, M.D., Lecturer on the Theory and Practice of Medicine in the Louisville Summer School of Medicine.

### LECTURE SECOND.

Before proceeding to a description of the physical signs of disease as derived from the conformation of the chest, or inspection, auscultation, and percussion, it will be proper to refer briefly to certain rules to be observed in the application of these different means of diagnosis.

1st. Inspection:—To inspect the chest to the best advantage, the patient should be in the erect position, arms hanging loosely by the side, and the chest entirely bare. It is customary with many physicians to make all examinations of this cavity through a thin garment. This, in the female, is to prevent exposure, and in the male to prevent the physician's face, in auscultating, from coming in contact with the filthy bodies of his patients. I have seen practitioners use for this purpose a silk handkerchief. To the use of any garment in inspection of the chest there is an insurmountable objection; it is not only necessary to examine its general conformation, but also the condition of each particular region: a garment however thin spread over the chest would only come in contact with the most projecting points, while the limited contractions or depressions, would entirely escape observation. From the condition of the intercostal spaces, we are sometimes enabled to distinguish between diseases of the thoracic cavity: if for example, with a certain group of physical signs, the intercostal spaces on one side, are bulging, beyond the level of the ribs, the disease is seated in the pleural cavity, either hydro-thorax or emphysema; but if with the same group of signs, these spaces be entirely natural, corresponding on the two sides, then the disease is confined to the substance of the lungs. The whole chest then, being entirely exposed, the physician can take in at a glance both sides, comparing the projections and depressions upon one side with the corresponding points upon the opposite side. The patient should be directed to take a deep inspiration, and then to breathe rapidly that the mobility of the chest may be tested also. In certain diseases patients breathe almost exclusively with the diaphragm and abdominal muscles, while in certain other diseases the respiratory movement is performed alone by the ribs. Any bending of the body either to the one side or the other, would interfere with the examination.

*Percussion.*—If the patient is unable to sit erect, care should be taken to have every thing moved from around the body, that would tend to obscure the sound; the bed-clothes must be removed; the body does not sink down in a matress as it does in a feather bed—the former is therefore preferable.

Percussion is divided into mediate and immediate; by mediate percussion is meant, striking the body through the medium of some instrument, which instrument is called a pleximeter; by immediate, striking directly upon the body with the fingers; the latter mode is never practised at the present day. The physician should so place himself that all his movements will be perfectly free, then adjusting the pleximeter accurately to the surface so that every part of it will be in contact with the body, it should be struck two or three times in rapid succession; by suffering the fingers to remain in contact with the instrument, the sound is obscured; the stroke should be given perpendicularly, and with the ends of the fingers. Percussion should be made alternately on the two sides, from the apex to the base of the chest, that you may have the advantage of comparison. The force to be used will depend upon the degree of muscular and adipose development over the organs to be examined; posteriorly it must be much more forcible than elsewhere, in consequence of the dense muscles and bone through which the sound must pass; a slight stroke would only elicit the dull sound of the muscles and bones, while a harder stroke would elicit a clear resonant sound from the organs within the cavity; in front, percussion must be a little more forcible in the precordial region where the lungs overlap the heart than elsewhere. It need never be sufficiently forcible to give pain.

*Auscultation.*—In auscultating the anterior portion of the chest, the body must be erect with the shoulders and arms thrown back, so that the muscles may be rendered tense and therefore better conductors of sound; in percussion, the soft parts may be pushed aside by the pleximeter, so that nothing but the integuments intervene between the instrument and the bony walls of the chest. In examining posteriorly and laterally, the position recommended in percussion is most favorable. The position of the patient is not more important than that of the physician; if he has to stoop with his head lower than his body, or bend over the patient in bed, the rush of blood to the head, or the irregular muscular contractions will produce certain sounds that may be confounded with those formed in the chest.

Auscultation like percussion is divided into mediate and immediate; mediate is the application of the ear through some medium to the body. Immediate is its direct application. The medium more generally employed is a small trumpet-shaped tube made of some light wood, called a stethescope. This mode is greatly preferable to immediate auscultation; 1st. That in applying the ear directly, the whole side of the face is brought in contact with the patient's body, and the hair rubbing against the body causes a friction sound not unlike those occasionally formed in the lungs. 2d. In the examination of negroes or of filthy whites, the direct application of the face would be very unpleasant. 3d. In the examination of females it is not practicable thus to apply the ear, for even if not restrained by feelings of delicacy the mammary glands would prevent it; by using the stethescope, however, these can be pushed aside, so as to allow the mouth of the instrument to be brought in contact with the walls of the chest proper; and lastly, *cæteris paribus*, the sound is rendered more intense, a larger number of vibrations are collected in the expanded extremity of the instrument and conveyed in a condensed form through the narrow extremity into the ear.

The instrument held between the thumb and middle finger so that they will at the same time be in contact with the chest, must be accurately adjusted. No opening must be allowed to exist between the mouth of the stethescope and the body of the patient; the sounds would not only escape, but extraneous sounds would be admitted. Then, having the ear-piece accurately adjusted to the ear, the auscultator must concentrate his whole attention upon the sounds.

Having now described the characters of the healthy sounds of respiration and the manner of examining the chest by inspection, auscultation, and percussion, I come next to speak of those sounds which are produced by disease.

In the study of the morbid respiratory sounds, those cases are preferable which present these sounds in the widest deviation from their normal characters; thus in the advanced stage of phthisis, in extensive and acute pneumonia, in pleurisy, with large deposition of lymph, and in acute and severe bronchitis, you have a group of symptoms so intense, that they can be distinctly heard by one who would fail entirely to appreciate the slighter alterations from the healthy state; still in describing these alterations I shall commence with the slightest.

The healthy respiratory sounds may either be altered in degree or changed in character by disease. In pneumonia a part of the lung being impervious to the air, the remaining portion compensates for it by taking on increased action; hence the sounds are louder in the healthy part, than they were previous to the attack of pneumonia; the same is true in regard to tubercular disease of the lungs, pleuritic effusions, in a word, to all diseases that impair the functions of any part of the lungs. If the disease be very extensive, this increased intensity may amount to the puerile respiration, a variety, as you know, healthy in the infant but diseased in the adult. The respiratory sounds are also diminished by disease; but this never occurs without an alteration in the relative loudness of the blowing and vesicular sounds. I am well aware that this opinion is at variance with the writers upon this subject, who teach that both sounds are lessened, equally, in emphysema of the lungs, and in pleuritic effusions. A moment's reference, however, to the anatomical structure of the lungs, will show the erroneousness of this opinion. The external or vesicular portion of the lung in which the vesicular murmur has its seat, is much more compressible than the internal or bronchial portion, where the blowing sound is formed; hence in emphysema, hydro-thorax, &c., the second sound is often obliterated entirely, while the first is heard distinctly, which certainly indicates that there is from the commencement of this effusion to the obliteration of the vesicles, a disproportion between the alterations in the two sounds. In emphysema, the disease is confined almost exclusively to the vesicles of the lungs, these are distended and relaxed; any alteration, therefore, in the respiratory sounds must be confined to that element which is formed in this diseased portion. While in emphysema then, the vesicular murmur is

1 *

not heard at all, the blowing sound is but little if at all changed. In that variety of emphysema common to old persons, the blowing sound is also very materially enfeebled, but this is doubtless owing to that general decay which is taking place in all the organs of the body, and the consequent diminished energy of their functions. Another class of alterations consist essentially in a want of proportion between the blowing and vesicular sounds; in this class are embraced rude respiration, bronchial respiration, tubal respiration, and cavernous respiration: another variety has been described, called the amphoric; this is but a variety of the cavernous, however, and may come very properly under that head.

*Rude Respiration.*—This consists in a feebleness of the vesicular murmur, with a slightly increased loudness of the blowing sound—both in inspiration and expiration—it occurs in consequence of the partial obliteration of the vesicular structure of the lung; in phthisis, for example, the tubercular deposit is at first very slight, causing a partial compression of the minute tubes and vesicles. The sound is therefore less in this compressed portion than natural, while the adjacent bronchial portion acts a little more energetically to compensate for the diminution in the compressed part; the bronchial or blowing sound is also increased from the tubercular deposit being a better conductor of sound than the healthy pulmonary structure.

*Bronchial Respiration.*—When the deposit increases so as to obliterate the vesicular portion of the lung entirely, the blowing sound is still farther increased, while the vesicular murmur is absent of course. The respiration being confined entirely to the bronchial tubes, is called bronchial respiration; any disease that will obstruct the passage of air into the vesicular portion of the lung will cause this sound.

*Tubal Respiration.*—This is a sound almost peculiar to pneumonia; it is heard when a large portion of the lung, including some of the larger bronchial tubes, is in a state of solidification. The only sound heard is that made by the passage of air through these tubes, the walls of which are solid and unyielding, it resembles that made by the blowing into a flute with all the holes closed, or into any other solid tube.

*Cavernous Respiration.*—As the name implies, this sound is formed in a cavity or cavern in the lungs, it is therefore almost peculiar to phthisis, although it may sometimes occur in pneumonia; here also the second sound is absent, while the first is very much changed in character. It resembles the sound made by blowing into a narrow necked vessel, the neck represented by the bronchial tube and the body by the cavern; the character of the sound is little changed with the size of the cavern, which has caused the addition of another sound before alluded to. To the production of the cavernous respiration it is essential that there be free communication with the bronchial tube, and that the cavity be empty. It is generally heard at the summit of the lungs—this being the point where tubercular deposit first occurs. This sound bears some resemblance to that last described; they can generally be distinguished by their locality. The tubal respiration always occurs over the region of the larger bronchial tubes, while, as before stated, the summit is the usual seat of the cavernous. Again, the tubal is heard over a much larger surface; the cavernous being in all cases limited to the part directly over the cavity.

*Rhonchi.*—The sounds which we have just described involve necessarily some change in the substance of the lung, while the rhonchi, with but one exception, depend upon disease in the mucous membrane lining the air passages. These sounds are much more easily distinguished than those which depend alone upon alteration in the natural respiratory sound. The rhonchi, rales or rattles, for these terms I shall use indifferently, are divided into the dry rhonchi, and the humid or wet rhonchi. The dry rhonchi are formed by the passage of air through the bronchial tubes in a state of inflammation, or when the mucous membrane is thickened, or coated by thick tenacious mucus, which is not dislodged during respiration; under this head there are two varieties, the sonorous rhonchus and the si blant rhonchus.

The sonorous rhonchus is produced by the friction of the air against the mucous membrane in a state of congestion and dryness; there are two conditions necessary to the production of this sound—diminution in the caliber of the tubes, and roughening of their surfaces. These conditions al-always exist in the first stage of acute bronchitis, in chronic bronchitis, when the lining membrane is thickened by deposits of lymph beneath it, and in some rare cases when the expectoration is so viscid as to adhere firmly to the sides of the tubes. This sound has been compared to the cooing of a dove, hence it is sometimes called the cooing sound; it resembles also very much the clear ringing noise made in snoring. It is formed in the larger bronchial tubes, and is heard both in inspiration and expiration.

*Sibilant Rhonchus.*—This only differs from the other in degree, it is a sharper, shriller sound, resembling a low whistle, and is formed in the smaller bronchial tubes, in the first stage of inflammation. The next rhonchi are formed by the passage of air through the bronchial tubes containing some fluid. They consist in the cavernous rhonchus, the mucous sub-crepitant, and crepitant.

The *Cavernous Rhonchus*, like the cavernous respiration, is formed in a cavity; but to produce the first, the cavern must be empty and to produce the last it must contain a fluid. This sound is sometimes called gurgling from its peculiar character; the word will give some idea of the sound. In almost all cases it occurs in cavities from tubercular excavations; hence its usual seat is in the summit of the lungs. It is caused by the passage of air through a thick homogeneous mass, in a circumscribed cavern.

*Mucous Rhonchus.*—This rhonchus is characterized by an irregular bubbling sound; when we blow through any hollow tube into a liquid, bubbles are produced, and the breaking of these bubbles is attended with a slight noise. The character of this noise will vary with the consistence of the liquid. In bronchitis when the inflammation is recent and not intense, a thin watery mucus is secreted; as it advances the fluid becomes whitish, opaque, and a little more tenacious; and still later it is much more tenacious and of a yellow color; the air passing through these different fluids, causes a succession of irregular bubbles extending over a very considerable surface, called the mucous rhonchus.

The *Sub-Crepitant* bears the same relation to this sound that the sibilant rhonchus does to the sonorous. It is formed in the smaller bronchial tubes and only differs from the mucous in degree. The bubbles are rather sharper; it requires a very practiced ear to distinguish between them.

*Crepitant Rhonchus.*—This sound is peculiar to pneumonia; it is formed by the passage of air through the minute tubes and vesicles of the lungs containing viscid mucus; it has been compared to the sound made by rubbing a lock of hair between the fingers close to the ear, or to the crackling of salt when thrown upon hot coals. It is generally mentioned as peculiar to the first stage; it may however be heard in the second or third stages, in the second or hepatizdd stage, this sound ceases where it was originally heard, but the presence of the diseased mass excites inflammation in the surrounding tissue, and in this the crepitation may be heard. The metallic tinkling or new-leather or creaking sound, does not come properly under the heads mentioned. The metallic tinkling is formed in a large cavity containing both air and liquid, and having also a communication with the external atmosphere. It may be produced in three ways; 1st, by a drop falling from the top of the cavity into the liquid beneath; 2d, by decomposition and disengagement of gas in the portion of the cavity beneath the liquid; and lastly, the air passing through a bronchial tube into this cavity below the level of the liquid.

*Voice.*—If the ear be applied to the chest of a healthy person while speaking, a confused roaring sound is heard; this is equal at all points, save the summit of the right lung, where it is naturally slightly louder than elsewhere, owing to the anatomical arrangement of the bronchi.

If the lungs become solidified at any point, the voice is much more distinctly heard there, as it is then a much better conductor of sound than in a healthy state when almost the entire bulk is made up of air. When tubercles are first deposited, a slightly increased loudness or resonance of the voice is heard at the point of deposite. This increase is not very great, however, as the solid matter is small, and the air still enters the minute bronchial tubes around which it exists.

If the solid matter exists in sufficient quantity to obliterate entirely the vesicular portion of the lungs, as in the more advanced stages of phthisis, or in pneumonia, &c., then this alteration in the voice is very great, and is called broncophony.

If a cavity exists in the lungs lined by a false membrane, and empty, not only is the intensity of the voice very great, but the articulation is also quite distinct; the sound seems in this case to come directly from the chest into the ear, as though one spoke into the mouth of the stethescope, while applied to the ear. It is called pectoriloquy. While the presence of this sound always indicates the existence of a cavity, its absence by no means proves that one does not exist, for if it contains mucus or pus, or broken down tubercular matter, pectoriloquy is not heard.

A circumscribed dilatation of the larger bronchial tubes may emit a sound very similar to this, but they may generally be distinguised by the locality. Pectoriloquy being heard almost uniformly at the summit of the lungs, where tuberculous excavations usually occur, while the sound emitted by a dilated bronchus would only be heard in the region of these tubes.

Amphoric resonance has been applied to an indistinct pectoriloquy extending over a larger surface, and formed by the excavation of an entire lobe of the lungs.

I shall notice but one other alteration of the voice—egophony. This is a tremulous vibratory sound, formed by the passage of the voice through a thin stratum of liquid, and is therefore heard in the first stage of pleuritic effusion.

